# Oral Leiomyoma in a Pediatric Patient: A Diagnostic Challenge

**DOI:** 10.7759/cureus.41107

**Published:** 2023-06-28

**Authors:** Amit Zope, Satyabrat Banerjee, Gargi Deshmukh, Junaid A Syed, Manish Sharma

**Affiliations:** 1 Orthodontics, JMF's (Jawahar Medical Foundation) ACPM (Annasaheb Chudaman Patil Memorial) Dental College, Dhule, IND; 2 Endodontics, JMF's (Jawahar Medical Foundation) ACPM (Annasaheb Chudaman Patil Memorial) Dental College, Dhule, IND; 3 Dental Surgery, Deshmukh Dental Clinic, Aurangabad, IND; 4 Orthodontics, Saraswati Dhanwantari Dental College, Parbhani, IND; 5 Oral Pathology and Microbiology, JMF's (Jawahar Medical Foundation) ACPM (Annasaheb Chudaman Patil Memorial) Dental College, Dhule, IND

**Keywords:** surgical excision, smooth muscle, immunochemical analysis, tongue, leiomyoma

## Abstract

A nodular lesion of the tongue incorporates a spectrum of entities from reactive to malignancy. A diagnostic dilemma arises when a nodular, solitary, and firm submucosal mass appears in the oral cavity of a patient. To reach a definitive diagnosis, a crucial investigation protocol needs to be followed. Leiomyomas are benign tumors that rarely occur in the oral cavity. They usually affect patients within the fourth to fifth decades of life. We report a rare case of leiomyoma of the tongue in a 12-year-old child patient. Immunohistochemical analysis revealed the positivity of tumor cells for alpha-smooth muscle actin (SMA). Surgical excision of such lesions is the treatment of choice, with a low recurrence rate. Our patient was asymptomatic and tumor-free at the follow-up visit after two years.

## Introduction

Leiomyomas are benign, well-circumscribed, soft-tissue tumors that arise from smooth muscles. The preliminary description of leiomyoma was given by Virchow in 1884. It mainly affects the uterus (95%), gastrointestinal tract (1.5%), and skin [1]. They are rarely found in the oral cavity, with an incidence of 0.42% [2]. In the oral cavity, it affects the tongue, palate, lips, and buccal mucosa. The origin of oral leiomyomas is hypothesized from smooth muscles, present in tunica media of blood vessels, ductus ligulas, and circumvallate papillae [3]. Oral leiomyomas are benign; they rarely undergo malignant transformation. Clinically, they present well-defined nodular submucosal masses, which are slow-growing and asymptomatic. It is usually seen in the fourth to fifth decades of life and affects males more than females [4]. They are asymptomatic, not often painful, with a smooth surfaced mass, a color similar to that of the adjacent mucosa, and slow growth. Due to their rare occurrence in the oral cavity and non-specific clinical presentation, the precise diagnosis is difficult. A histological examination and immunohistochemical analysis play a very important role. Histologically, they present spindle-shaped cells arranged in interwoven fascicles or whorled patterns. The World Health Organization has classified oral leiomyomas into three histological grades: solid, vascular, and epithelioid [2]. Surgical excision of the tumor mass is the treatment of choice, with a low recurrence rate [5]. The objective of this case report was to present a rare case of leiomyoma of the tongue in a 12-year-old male child who was tumor-free at a follow-up visit of two years.

## Case presentation

**Figure 1:**
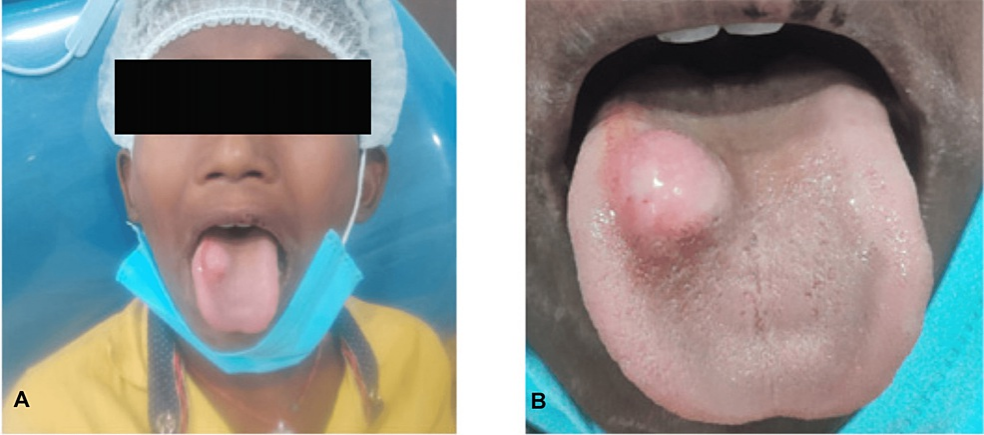
Solitary, well-circumscribed, nodule (A) Solitary, well-circumscribed, nodule on dorsum surface of tongue. (B) Firm swelling of size approximately 2×1.5 cm having a smooth surface.

A 12-year-old male child presented to the Department of Oral and Maxillofacial Surgery with a swelling of the tongue for one year. The patient reported no remarkable medical, dental, or habit history. No record of trauma or tongue bite was observed. Extraoral examination results were normal. Intraorally, a solitary, asymptomatic (no pain and tenderness), well-circumscribed, firm, nodular swelling of size approximately 2×1.5 cm was present on the mid-dorsal surface of the tongue on the right side (Figure 1A). It had the same color as normal mucosa. The nodule was sessile, submucosal, smooth-surfaced, and freely movable. No bruit or pulsation was noticed at the lesional site (Figure 1B).

**Figure 2:**
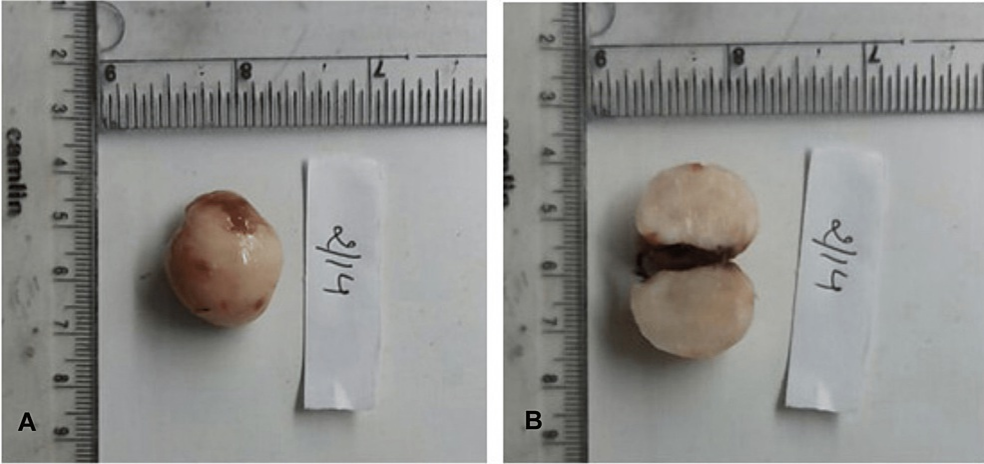
Gross examination of the specimen (A) Well-circumscribed excised lesion. (B) Cut surface of the lesion is whitish without any cystic component.

There was no increase in the size of the nodule since it was first noticed. The ultrasonographic examination revealed the absence of any calcification or noticeable findings of hemangiomas and lipomas. Fine needle aspiration cytology (FNAC) investigation was also negative as no fluid or blood content was retrieved, which ruled out the hemorrhagic origin of the lesion. No signs of paresthesia were found. Clinical and preoperative findings clarified the benign nature of the lesion. Based on the clinical and radiographic investigations, a provisional diagnosis of solitary fibrous tumor, neurofibroma, and schwannoma was made. Taking into account the age and size of the lesion, the mass was excised under local anesthesia. Because the mass was well-circumscribed, an elliptical incision was made, and the lesion was completely enucleated with the surrounding mucosa to prevent recurrence (Figure 2A). On gross examination, it was oval, firm, well encapsulated, and pinkish-white in color, and the cut surface showed discrete necrosis of the tissue (Figure 2B).

**Figure 3:**
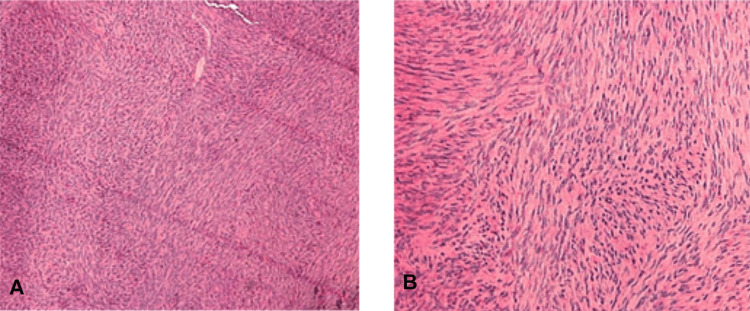
H&E-stained section (A) H&E-stained section at 4× magnification shows an ill-defined lesional area having rich cellular component. (B) H&E-stained section at 10× magnification shows fascicles of spindle cells. H&E: hematoxylin and eosin.

Histological examination at 10× magnification (hematoxylin and eosin stain) revealed spindle-shaped cells arranged in irregular fascicles (Figure 3A) and a whorled pattern of cells with blunt nuclei (Figure 3B).

**Figure 4:**
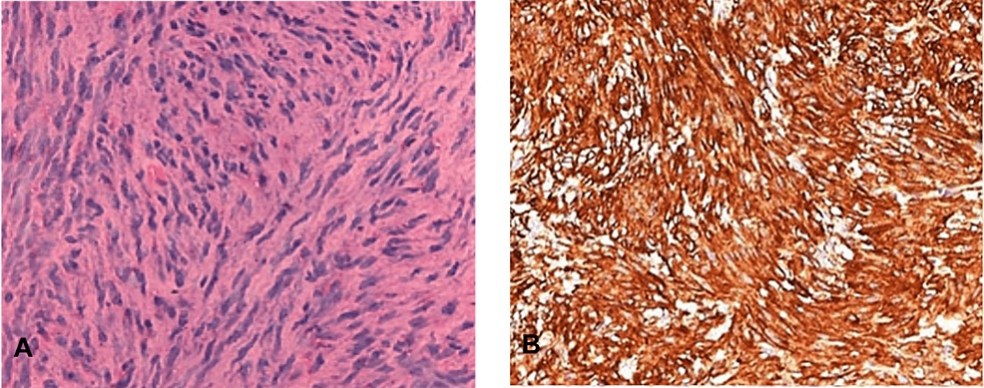
At 40× magnification (A) Hematoxylin and eosin (H&E)-stained section at 40× magnification showing vesicular nuclei of spindle cells. (B) Smooth muscle actin-rich section at 40× magnification.

At 40× magnification, spindle cells showed ill-defined cytoplasm and mildly pleomorphic vesicular nuclei (Figure 4A). Abnormal mitosis was not found in any microscopic field which ruled out a malignancy. Immunohistochemistry results showed intense cellular staining for the smooth muscle actin (Figure 4B) confirming the smooth muscle origin of the lesion.

Based on these findings, a definitive diagnosis of oral leiomyoma was made. Due to the financial constraint of the patient, further investigations were not conducted. On follow-up visits for two years, the patient was asymptomatic and free of tumor after surgical excision of the lesion.

## Discussion

A solitary nodule of the tongue with a smooth surface can clinically come under the differential diagnosis of fibrous hyperplasia, schwannoma, neurofibroma, lipoma, hemangioma, leiomyoma, and salivary gland tumors. [6]. A precise diagnosis can be achieved by routine clinical investigations followed by other necessary laboratory parameters. Leiomyomas of the oral cavity are theorized to originate from smooth muscle present in the ductus lingualis, circumvallate papillae, or tunica media of blood vessels, as evident from the previous literature [7,8].

A debatable pathogenesis of leiomyoma is available in the literature, although traumatic injury, venous stasis, hormonal alterations, and genetic changes can be considered. They usually occur in the fourth and fifth decades of life and on the lips, buccal mucosa, or tongue [9]. In our case, it was noticed in a 12-year-old boy on the right dorsal surface of the tongue. Although leiomyomas are rare in a pediatric population, yet they can mimic schwannoma or fibrous hyperplasia. The literature showed that the incidence of age varied from two months to 85 years [10].

Most of these lesions are asymptomatic and slow growing, as in our case, but some authors have reported symptomatic findings, such as difficulty in chewing and swallowing due to the presence of a tumor mass [11]. Our patient had a tumor mass for one year; it was a well-defined, smooth surface, without any change in color, firm, sessile, and submucosal. Previous literature showed that the clinical appearance of leiomyoma may vary according to the intraoral location; it may appear as a mucocele on the lip and a vascular lesion on the gingiva [12].

Histologically, it was a solid type leiomyoma that was rare in the oral cavity, comprising spindle-shaped cells interwoven in a whorled pattern with collagen strands in-between. All histological characteristic findings of solid leiomyoma were present in our case [13]. Other spindle cell tumors like solitary fibrous tumors, neurofibroma, and schwannoma should be ruled out by histopathological features and immunohistochemical analysis. The present case was positive for smooth muscle actin (SMA), but neurofibroma and schwannoma show positivity with S100 and the solitary fibrous tumor shows spindle cells reactive to the CD34 marker. Therefore, a thorough routine histopathological examination and immunochemistry analysis are required for accurate diagnosis. The treatment of oral leiomyoma involves surgical excision, with a low recurrence rate; in our case, the patient was reviewed for two years, and no recurrence was observed.

## Conclusions

The present case was reported to improve our understanding of leiomyoma, although it is a rare entity in the oral cavity but should not be neglected in pediatric patients. Due to its rare occurrence in the oral cavity and indistinct clinical findings, a thorough routine histological examination with immunochemical staining is demanded. The treatment selection is always considering the site and size of the lesion, although complete surgical excision is warranted.
